# Retinal Drusen Are More Common and Larger in Systemic Lupus Erythematosus With Renal Impairment

**DOI:** 10.1016/j.ekir.2022.01.1063

**Published:** 2022-02-02

**Authors:** Ye Ji Ham, Eleanor Nicklason, Tony Wightman, Sarah Akom, Kieran Sandhu, Philip Harraka, Deb Colville, Andrew Catran, David Barit, David Langsford, Tim Pianta, Andrew Foote, Russell Buchanan, Heather Mack, Judy Savige

**Affiliations:** 1 The University of Melbourne Department of Medicine, Northern Health and Melbourne Health, Melbourne, Australia; 2 Renal Unit, Northern Health, Melbourne, Australia; 3 Rheumatology Unit, Northern Health, Melbourne, Australia; 4 The University of Melbourne Department of Ophthalmology, The Royal Victorian Eye and Ear Hospital, East Melbourne, Australia

**Keywords:** complement, drusen, immune complexes, impaired kidney function, lupus nephritis, SLE

## Abstract

**Introduction:**

Complement has been implicated in systemic lupus erythematosus (SLE) pathogenesis on the basis of the associations with inherited complement defects and genome-wide association study risk alleles, glomerular deposits, reduced serum levels, and occasional reports of retinal drusen. This study examined drusen in SLE and their clinical significance.

**Methods:**

This cross-sectional observational study compared individuals with SLE recruited from renal and rheumatology clinics with hospital controls. Participants were reviewed for clinical features and underwent imaging with a nonmydriatic retinal camera. Deidentified images were examined by 2 trained graders for drusen number and size using a grid overlay.

**Results:**

The cohort with SLE (*n =* 65) comprised 55 women (85%) and 10 men (15%) with a median age of 47 years (interquartile range 35–59), where 23 (35%) were of southern European or Asian ancestry, and 32 (49%) had biopsy-proven lupus nephritis. Individuals with SLE had higher mean drusen numbers than controls (27 ± 60, 3 ± 9, respectively, *P* = 0.001), more drusen counts ≥10 (31, 48% and 3, 5%, respectively, *P* < 0.001), and more medium-large drusen (14, 22% and 3, 5%, respectively, *P* < 0.001). In SLE, mean drusen counts were higher, and drusen were larger, with an estimated glomerular filtration rate (eGFR) <90 ml/min per 1.73 m^2^ (*P* = 0.02, *P* = 0.02, respectively) or class IV nephritis (*P* = 0.03, *P* = 0.02).

**Conclusion:**

Drusen composition resembles that of glomerular immune deposits. CFH controls complement activation in the extracellular matrix and *CFH* risk variants are shared by drusen in macular degeneration and by SLE. CFH represents a possible treatment target for SLE especially with renal impairment.

## Graphical abstract

**Figure:**
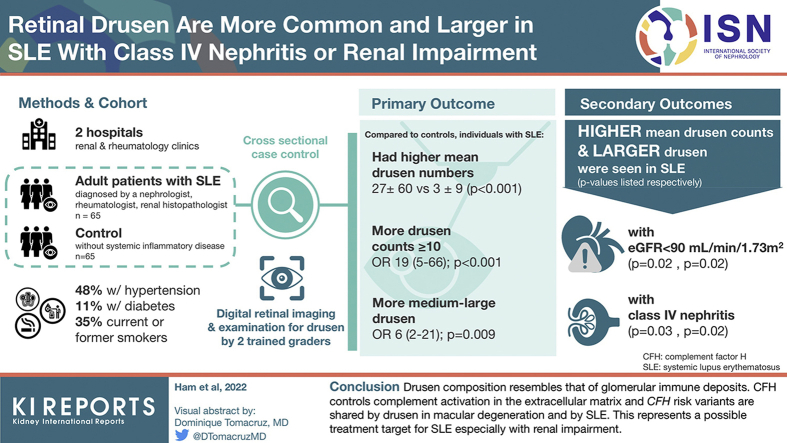

SLE is an autoimmune disease that affects 1 in 2000 individuals, mainly women, and is more common in people of Southern European, east Asian, and African ancestries.^1^ The diagnosis of SLE depends on the presence of an antinuclear antibodies, distinctive clinical features (constitutional, hematologic, neuropsychiatric, mucocutaneous, serosal, musculoskeletal, renal) and immunologic criteria (antiphospholipid antibodies, complement levels, SLE-specific antibodies).^2^ Renal involvement is common and varies from urinary sediment abnormalities or abnormal glomerular histology^3^ through to end-stage kidney failure.^4^ Renal involvement is classified histologically (ISN/Renal Pathology Society system): Class I and II disease is characterized by mesangial immune deposits, class III and IV disease by subendothelial and mesangial deposits, and class V by a membranous nephritis with subepithelial deposits. Class IV and V disease have a particularly poor prognosis, with 50% and 20% of affected individuals, respectively, reaching end-stage renal failure after 15 years.^5^

SLE is associated with activation of the classical complement cascade.^3^ Complement involvement in SLE pathogenesis is suggested by the association with inherited complement defects,^6^ reduced circulating complement levels in active disease, glomerular C3 deposits in lupus nephritis, genome-wide association studies, animal models, and the finding of retinal drusen.^3^^,^^7^, ^8^, ^9^ SLE is more common in individuals with inherited deficiencies of the classical complement pathway proteins C1q, C2 and C4. In active SLE, serum C3 and C4 levels are reduced.^7^ Glomerular immune complex deposition results in local C3 and C1q fixation and subsequent inflammation and tissue damage. Genome-wide association studies have implicated many complement pathway genes encoding proteins, receptors, and regulators (CD55, CD59, CD46, CD35, CFH),^9^ and complement pathway activation has been confirmed in mouse models of SLE.^10^

In addition, retinal drusen have been described in SLE.^11^, ^12^, ^13^, ^14^, ^15^, ^16^ Drusen are yellow-white deposits, visible on ophthalmoscopy and in retinal images, where up to 10 drusen occur in normal middle-age but increased numbers are found in age-related macular degeneration. Drusen comprise cell debris, extracellular matrix, immunoglobulins, complement, and C-reactive protein.^17^ Risk factors include genetics, age, hypertension, smoking, diabetes, and renal failure.^18^ Drusen have also been described in 40% of individuals with SLE and are more common, larger, and more widely distributed in the retina when there is renal involvement but, to date, have not been associated with renal impairment or a histologic disease type.^11^

The genetics of drusen development in macular degeneration is complex.^19^ More than 30 risk genes have been identified encoding proteins involved in the complement pathways, lipid metabolism, extracellular matrix metabolism, reactive oxidation, apoptosis, and angiogenesis.^20^, ^21^, ^22^ Complement involvement is critical. The drusen in macular degeneration arise from the inability to clear retinal pigment epithelial cell debris, where the membrane lipid activates complement. Together, the genes for *CFH*^23^^,^^24^ and *ARMS2/HTRA1*^25^ (age-related macular degeneration gene/high temperature requirement A-1) account for half the genetic risk for drusen.^19^ The abnormal CFH reduces complement inactivation and increases membrane attack complex activity and damage to the retinal pigment epithelium. The commonest *CFH* variant, Y402H, is associated with increased drusen number, and the homozygous form with more extensive retinal disease.^26^ *ARMS2/HTRA1* encodes a serine protease that degrades the extracellular matrix and possibly activates the alternative pathway regulatory complement factors B and D.^27^

Drusen have also been described in many other forms of glomerulonephritis (GN), namely, dense deposit disease, membranous and poststreptococcal GN,^11^^,^^28^ and atypical haemolytic uremic syndrome,^29^^,^^30^ which are all associated with risk alleles in *CFH* or other complement pathway genes (*CFH, CFI, C3)* and with complement activation.^31^ The composition of drusen in macular degeneration resembles that of the glomerular immune deposits in at least membranous and poststreptococcal GN.^32^

This study examined individuals with SLE to confirm that drusen were more common than in controls and to determine their clinical significance.

## Methods

### Study Design

This was a cross-sectional observational case-control study of consecutive individuals with SLE recruited over two 6-month periods from the renal and rheumatology clinics of a teaching hospital. Recruitment, data capture, and retinal photography were coordinated in a single episode. Retinal images were then examined for drusen by 2 trained graders.

The primary outcome was to confirm that drusen were more common in SLE than in matched controls, and the secondary outcomes were to determine whether drusen were associated with longer disease duration, with lupus nephritis or with renal impairment. There were no changes to the study design after its commencement and no interim analyses.

Inclusion criteria were age ≥18 years and a diagnosis of SLE made by a specialist nephrologist, rheumatologist, or renal histopathologist according to European League Against Rheumatism/American College of Rheumatology criteria, or renal biopsy features.^2^^,^^8^ Exclusion criteria were ungradable retinal images.

This project was approved by the Northern Health Human Research Ethics Committee, and written, informed consent was obtained from study participants according to the principles of the Declaration of Helsinki.

### Participants

Participants provided a brief medical history (age, sex, ancestry, disease duration, renal transplant status) and drusen risk factors (smoking, hypertension, diabetes), and their charts were reviewed for current eGFR measurements and renal biopsy results. Lupus nephritis class and glomerular complement staining were recorded: moderate or strong staining was considered positive; and weak or negative staining negative.

Controls were age- and gender-matched individuals without systemic inflammatory disease recruited from general medical or preoperative surgical clinics during the same time period.

### Measurements

#### Retinal Imaging and Grading for Drusen

**Figure 1:**
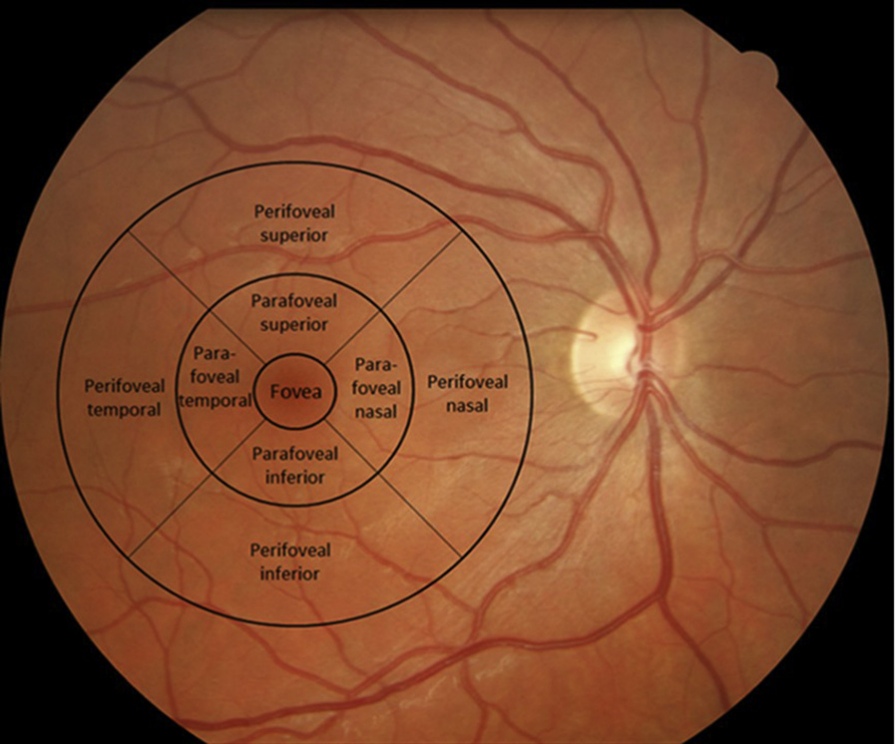
Drusen grading schemes for the macula, according to the modified ETDRS Grid. ETDRS, Early Treatment of Diabetic Retinopathy Study.

All participants underwent digital retinal imaging of both optic fundi with a nonmydriatic retinal camera (Canon CR5-45, Japan). Standard images were taken centered on the macula and optic disc of each eye. Deidentified images were examined for drusen by 2 trained graders, drusen were counted, using a grid overlay corresponding to the Wisconsin Age-Related Maculopathy Grading System^33^, and the numbers confirmed independently by an ophthalmologist (Figure 1). This method was highly reproducible, with an intraobserver interassay coefficient of variation of 18%.

Drusen counts in the central fovea and parafovea were recorded from the eye with the higher number. Counts ≥10 were considered abnormal.^34^ The numbers in the periphery were also counted where the periphery was considered to be at least 2 disc diameters from the central macula.

Drusen size was assessed by comparison with the span of the largest venule where it crossed the disc margin (63 μm) as small (<63 μm), medium (63–125 μm), or large (>125 μm).^35^ Drusen may be complicated by overlying retinal atrophy or pigmentation, and these were recorded independently by an ophthalmologist.

### Statistical Analyses

Categorical variables, including demographic, clinical, and drusen characteristics were summarized as percentages and compared using the Fisher exact test. Continuous variables were compared with *t* test, or the Mann-Whitney *U* test if non-normally distributed. Odds ratios and 95% CIs were calculated with univariate logistic regression. Analyses were performed using SPSS (IBM, Armonk, NY). A *P* < 0.05 was considered significant, and a value between 0.05 and 0.10 was considered a trend.

## Results

### Clinical Features

Sixty-five participants with SLE were recruited, after 3 were excluded because their retinal images were ungradable because of cataracts (Table 1). They comprised 55 women (85%) and 10 men (15%) with a median age of 47 years (interquartile range 35 to 59). Forty-two (65%) were of Northern European and 23 (35%) of Southern European or Asian ancestry. A total of 31 (48%) had hypertension, 23 (35%) were current or former smokers, and 7 (11%) had diabetes.

**Table 1:** Characteristics of participants with SLE and hospital controls

Characteristics	SLE (*n =* 65)	Controls (*n =* 65)	*P* value, OR (95% CI)
Age (median, IQR, yr)	47 (35 – 59)	47 (35 – 67)	0.32
Female, *n* (%)	55 (85)	55 (85)	1.00 (0.39–2.59), *P* = 1.00
Ethnicity			
Northern European, *n* (%)	42 (65)	44 (68)	0.87 (0.42–1.80), *P* = 0.92
Southern European and Asian, *n* (%)	23 (35)	21 (32)	
Risk factors for drusen			
Hypertension, *n* (%)	31 (48)	19 (29)	**2.21 (1.07–4.54), *P* = 0.03**
Smoking history, *n* (%)	23 (35)	29 (45)	0.68 (0.34–1.48), *P* = 0.28
Diabetes, *n* (%)	7 (11)	6 (9)	1.19 (0.38–3.75), *P* = 0.77
SLE			
Disease duration ≥5 yr, *n* (%)	26 (50)		
Lupus nephritis, *n* (%)	32 (49)		
eGFR, median, IQR, (ml/min/1.73 m^2^)	70 ± 27	85 ± 10	***P* < 0.0001**
eGFR <90 (ml/min per 1.73 m^2^), *n* (%)	36 (55)	22 (34)	**2.17 (1.07–4.43), *P* = 0.03**
ESKF eGFR ≤15, *n* (%)	11 (17)	0	
Drusen number and location, *n* (%)			
Any drusen	50 (77)	29 (45)	**4.14 (1.94–8.82), *P* < 0.001**
Mean drusen number (SD)	**27 (60)**	**3 (9)**	***P* = 0.001**
≥10 drusen	31 (48)	3 (5)	**18.84 (5.36–66.21), *P* < 0.001**
Foveal	35 (54)	11 (17)	**5.73 (2.55–12.89), *P* < 0.001**
Drusen in ≥4 central areas	27 (42)	3 (5)	**14.68 (4.17–51.73), *P* < 0.001**
Bilateral	30 (51)	12 (21)	**3.79 (1.71–8.38), *P* = 0.001**
Medium drusen, *n* (%)	14 (22)	3 (5)	**5.67 (1.54–20.83), *P* = 0.009**
Large drusen*, n* (%)	8 (12)	0	**11.9 (0.65–219.96), *P* = 0.096**
Atrophy, *n* (%)	8 (12)	0	**19.37 (1.09–342.96), *P* = 0.04**
Pigmentation, *n* (%)	1 (2)	0	3.05 (0.12–76.18), *P* = 0.50

eGFR, estimated glomerular filtration rate; ESKF, end-stage kidney failure; IQR, interquartile range; OR, odds ratio; SLE, systemic lupus erythematosus.

ESKF: eGFR ≤15 ml/min per 1.73 m^2^, dialysis, or renal transplantation.

Significant values are indicated in bold.

Twenty-six individuals with SLE had a disease duration of at least 5 years (52%). A total of 32 (49%) had biopsy-proven lupus nephritis (49%) (Table 1). Their mean eGFR was 70 ± 27 ml/min per 1.73 m^2^, 36 (55%) had an eGFR <90 ml/min per 1.73 m^2^, and 11 (17% had reached end-stage kidney failure.

Renal biopsy reports were available for 24 of those with lupus nephritis. One (4%) had a tubulointerstitial nephritis with class I disease, 6 (25%) had class III, 9 (38%) had class IV, 3 (13%) had class IV/V, and 5 (21%) had class V lupus nephritis. C3 staining was positive in 13 (13 of 20, 65%), and C1q staining in 16 (16 of 20, 80%).

Individuals with SLE were different from the age- and gender-matched controls only in that they were more likely to have hypertension (*P* = 0.03) and a lower mean eGFR (*P* = 0.0001) (Table 1).

### Drusen Numbers, Clusters, Size, and Complications

None of the SLE cohort had any of the characteristic lupus retinal features such as choroidoretinitis or severe hypertensive changes.

**Figure 2:**
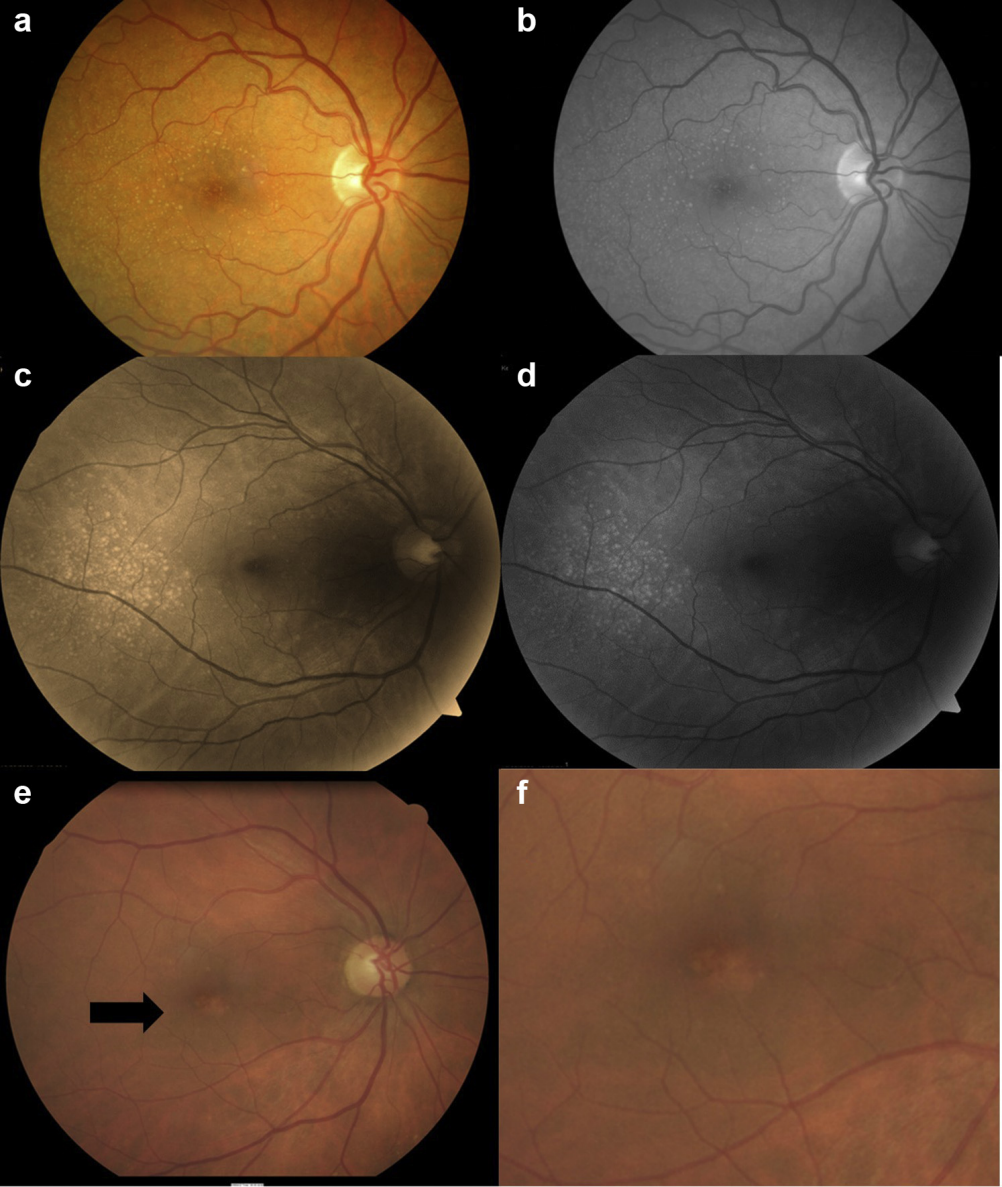
Retinal images demonstrating drusen in SLE. (a) An example of numerous retinal drusen in an individual with SLE; (b) black and white image of a. demonstrating the drusen more clearly; (c) drusen at the temporal macular in an individual with class IV nephritis; (d) black and white image of c. demonstrating the drusen more clearly; (e) retinal pigment epithelial atrophy and depigmentation (arrow) in an individual with SLE; and (f) enlarged view of the retinal atrophy. SLE, systemic lupus erythematosus.

Individuals with SLE were more likely to have drusen (*P* < 0.001), higher drusen counts (27 ± 60 and 3 ± 9, *P* = 0.001), more counts ≥10 (*P* < 0.001), drusen in all 4 central quadrants (*P* < 0.001), and drusen that were bilateral (*P* = 0.001), medium or large (*P* = 0.009), or associated with retinal atrophy (*P* = 0.04) than controls (Table 1 and Figure 2).

Individuals with SLE and medium or large drusen had higher mean total drusen counts than those with only small drusen (64 ± 110, *n =* 14 and 22 ± 49, *n =* 51, *P* = 0.04).

A total of 4 individuals (13%) with lupus nephritis also had peripheral clusters of >30 drusen mainly in the temporal retina (Figure 2). All 4 were female, aged from 23 to 63 years, 2 of Southern European ancestry, diagnosed from 1 to 8 years previously, and with kidney function ranging from normal to kidney failure. They included 2 with class IV lupus nephritis, and 3 of the 4 had large drusen.

One individual underwent further studies with optical coherence tomography, which demonstrated that the drusen were located beneath the retinal pigment epithelium and disrupted Bruch’s membrane.

Drusen in SLE were not more common or larger with the typical drusen risk factors of hypertension, smoking, or diabetes. Although the cohort with SLE had more hypertension than controls it may still have been too small to demonstrate a difference in drusen counts.

Drusen were associated with retinal atrophy in 8 individuals with SLE (12%), in all cases with more abundant or larger drusen, and no atrophy was found in the controls (*P* = 0.04). Pigmentation was present in 1 individual with SLE (2%) and no controls (*P* = 0.50).

### Drusen in SLE

In the cohort with SLE (*n =* 65), drusen counts were higher in women (*P* < 0.04), people aged >40 years (*P* < 0.03), or with renal impairment (*P* < 0.02) (Table 2).

**Table 2:** Drusen number, counts ≥10, and size in all participants with SLE

All SLE-characteristics (*n* *=* 65)	Drusen number, mean (SD)	*P* value	≥10 drusen, *n* (%)	OR, 95% CI, *P* value	Medium or large drusen, *n =* 14, *n* (%)	OR, 95% CI, *P* value
Age						
≥40 yr (*n =* 43)	36 (71)	**0.03**	24 (56)	2.21 (0.77–6.36), *P* = 0.14	12 (28)	**3.87 (0.78–19.15), *P* = 0.097**
<40 yr (*n =* 22)	10 (20)		8 (36)		2 (9)	
Sex						
Female (*n =* 55)	30 (10)	**0.04**	26 (47)	3.59 (0.70–18.44), *P* = 0.13	13 (24)	2.79 (0.32–24.10), *P* = 0.35
Male (*n =* 10)	12 (8)		6 (60)		1 (10)	
Ethnicity						
Southern European or Asian (*n =* 23)	28 (49)	0.93	16 (70)	**3.71 (1.25–10.99), *P* = 0.02**	8 (15)	**3.20 (0.95–10.81), *P* = 0.06**
Northern European (*n =* 42)	27 (66)		16 (38)		6 (14)	
Risk factors for drusen						
Hypertension (*n =* 31)	31 (69)	0.38	15 (48)	0.94 (0.35–2.48), *P* = 0.90	7 (23%)	1.13 (0.34–3.67), *P* = 0.85
No hypertension (*n =* 34)	17 (49)		17 (50)		7 (21%)	
Smoking (*n =* 23)	24 (57)	0.79	10 (43)	0.70 (0.25–1.95) *P* = 0.49	3 (13%)	0.42 (0.10–1.71), *P* = 0.23
Nonsmokers (*n =* 42)	29 (62)		22 (52)		11 (26%)	
Diabetes (*n =* 7)	12 (25)	0.92	1 (14)	**0.15 (0.01–1.28) *P* = 0.05**	1 (14)	0.58 (0.06–5.23), *P* = 0.49
No diabetes (*n =* 58)	13 (38)		31 (53)		13 (22)	
Disease duration						
≥5 yr (*n =* 26)	24 (56)	0.90	15 (58)	2.27 (0.73–7.07), *P* = 0.15	7 (27)	1.19 (0.42–3.43), *P* = 0.75
<5 yr (*n =* 24)	22 (30)		9 (38)		4 (17)	
Presence of lupus nephritis						
Lupus nephritis (*n =* 32)	24 (46)	*P* = 0.70	15 (47)	0.94 (0.35–2.48), *P* = 0.89	4 (13)	1.03 (0.23–4.55), *P* = 0.96
No lupus nephritis (*n =* 33)	30 (71)		16 (48)		4 (12)	

OR, odds ratio; SLE, systemic lupus erythematosus.

Significant values are indicated in bold.

Abnormal counts of at least 10 drusen were more common in people of Southern European or Asian ancestry (*P* = 0.02), or with renal impairment (*P* = 0.03).

Medium or large drusen were more common in people aged >40 years (*P* = 0.09), of Southern European or Asian ancestry (*P* = 0.06), with renal impairment (*P* = 0.02), or class IV nephritis (*P* = 0.02). However, drusen number and size were not associated with end-stage kidney failure (both *P* nonsignificant).

### Drusen in Lupus Nephritis

In the cohort with lupus nephritis (*n =* 32), drusen counts were higher in people aged >40 years (*P* < 0.045), with longer disease duration (*P* = 0.03), renal impairment (*P* = 0.02), or class IV nephritis (*P* = 0.03) and demonstrated a trend with C3 deposits (*P* = 0.098) (Table 3). Abnormal counts of at least 10 drusen were more common in people aged >40 years (*P* = 0.03), of Southern European or Asian ancestry (*P* = 0.04), with longer disease duration (*P* = 0.02), or renal impairment (*P* = 0.03).

**Table 3:** Drusen number, counts ≥10, and size in participants with lupus nephritis

Characteristics	Drusen count, mean (SD)	*P* value	≥10 drusen, *n* (%)	OR, 95% CI, *P* value	Medium or large drusen, *n* (%)	OR, 95% CI, *P* value
Age						
≥40 yrs (*n =* 19)	34 (55)	**0.045**	12 (63)	**5.71 (1.16–28.07), *P* = 0.03**	7 (37)	**7.00 (0.74–65.94), *P* = 0.09**
<40 yrs (*n =* 13)	11 (26)		3 (23)		1 (8)	
Sex						
Female (*n =* 27)	26 (50)	0.35	11 (41)	0.17 (0.01–1.75), *P* = 0.14	7 (26)	1.40 (0.13–14.74), *P* = 0.78
Male (*n =* 5)	15 (13)		4 (80)		1 (20)	
Ethnicity						
Southern European or Asian (*n =* 11)	45 (67)	0.15	8 (73)	**5.33 (1.07–26.61), *P* = 0.04**	5 (46)	**5.00 (0.91–27.47), *P* = 0.06**
Northern European (*n =* 21)	13 (28)		7 (33)		3 (14)	
Disease duration						
≥5 yr (*n =* 13)	29 (38)	**0.03**	9 (69)	**7.5 (1.31–43.02), *P* = 0.02**	6 (46)	**11.14 (1.11–112.02), *P* = 0.04**
<5 yr (*n =* 14)	13 (34)		3 (21)		1 (7)	
Impaired renal function						
eGFR < 90 ml/min/1.73 m^2^ (*n =* 21)	35 (55)	**0.02**	13 (62)	**7.31 (1.25–42.81), *P* = 0.03**	8 (38)	14.48 (0.75–279.09), *P* = 0.08
eGFR ≥90 ml/min/1.73 m^2^ (*n =* 11)	4 (6)		2 (18)		0	
Renal transplant						
Renal transplant (*n =* 8)	21 (32)	0.74	5 (63)	2.33 (0.45–12.09), *P* = 0.31	2 (25)	1.00 (0.16–6.35), *P* = 1.00
No transplant (*n =* 24)	26 (51)		10 (42)		6 (25)	
Class of lupus nephritis						
Class IV (*n =* 9)	69 (72)	**0.03**	6 (67)	3.60 (0.62–21.03), *P* = 0.15	5 (56)	**16.25 (1.44–183.10), *P* = 0.02**
Non-class IV (*n =* 14)	6 (6)		5 (36)		1 (7)	
Glomerular complement deposition						
C3 deposition (*n =* 13)	33 (61)	0.098	6 (46)	1.14 (0.18–7.28), *P* = 0.89	2 (17)	1.09 (0.08–14.66), *P* = 0.95
No C3 (*n =* 4)	0.5 (0.6)		3 (43)		1 (14)	
C1q deposition (*n =* 16)	33 (61)	0.11	7 (44)	0.78 (0.09–6.98), *P* = 0.82	2 (14)	0.43 (0.03–6.41), *P* = 0.54
No C1q (*n =* 4)	6 (5)		2 (50)		1 (25)	

eGFR, estimated glomerular filtration rate; OR, odds ratio.

Significant values are indicated in bold.

Drusen occurred in all classes of lupus nephritis, with mean counts of 3 ± 5 in class III (*n =* 6), 68 ± 72 in class IV (*n =* 9), 7 ± 6 in class IV/V (*n =* 3) and 9 ± 7 in class V (*n =* 5). Thus counts in class IV nephritis were higher than in class III (*P* = 0.03), class IV/V (*P* = 0.04), and class V (*P* = 0.04).

Drusen were also present in an individual with tubulointerstitial nephritis and C3 staining but not GN.

Medium or large drusen demonstrated trends or were more common in people aged >40 years (*P* = 0.09), of Southern European or Asian ancestry (*P* = 0.06), with longer disease duration (*P* = 0.04), renal impairment (*P* = 0.08), or class IV nephritis (*P* = 0.02).

## Discussion

Retinal drusen are common in SLE occurring in nearly half of all affected individuals. Drusen are more abundant and larger in SLE, especially in women, and people aged >40 years, or of Southern European or Asian ancestry. Drusen are also more common and larger in people with SLE with longer disease duration, renal impairment, or class IV nephritis. Drusen in SLE are not, however, associated with an increased risk of renal involvement or of end-stage kidney failure.^11^

In this cohort with SLE, drusen were found throughout the central macula but especially in the temporal quadrant. This distribution may be explained by the thinned central and temporal retina that is able to accommodate bulkier deposits, or from limitations from the glial cell distribution or blood supply.^11^ Multiple drusen were also sometimes present in the peripheral retina but occurred too rarely to demonstrate significance. In addition, drusen were found in lupus-associated tubulointerstitial nephritis without glomerular disease, which in a murine model of SLE is associated with complement activation.^10^

The increased drusen count found in this cohort and previously^11^ may have underestimated the actual number because retinal imaging is relatively insensitive in detecting drusen. Instead, the results may mean that nearly half the individuals with SLE had drusen large enough to be visible on retinal imaging and, thus, that the clinical associations were for drusen large enough to be visualized. The correlation of higher drusen counts and larger size in the same individuals was consistent with larger drusen resulting from the accretion of smaller forms or from a persisting stimulus for drusen enlargement. It was unclear how soon after the disease onset that drusen were first evident, whether they were resorbed with treatment, and, thus, whether drusen number and size reflected disease activity or rather disease duration.

The appearance and location of drusen in SLE resembles those for macular degeneration, but the drusen were smaller and the individuals too young. In addition, the drusen in SLE were not explained by the traditional macular degeneration risk factors of hypertension, smoking, or diabetes. Although macular degeneration is also more common in renal impairment,^18^ drusen were commonly associated with normal kidney function in this cohort.

Indeed, drusen have been described in other forms of GN such as C3 glomerulopathy, dense deposit disease, and IgA GN,^11^^,^^28^, ^29^, ^30^^,^^36^^,^^37^ but these diseases had already been excluded by renal biopsy. Retinal abnormalities with a similar appearance including hard exudates, inherited retinal dystrophies (Sorsby’s macular dystrophy, Malattia Leventinese, pattern macular dystrophy), the Alport fleck retinopathy, and epiretinal membranes were excluded by a retinal expert.

The larger drusen in SLE were sometimes associated with retinal atrophy, but atrophic areas were too small to affect vision.^38^ This contrasts with C3 glomerulopathy and dense deposit disease, where extensive drusen result in visual loss from retinal atrophy, pigmentation, edema, hemorrhage, and neovascularization.^39^^,^^40^

Drusen potentially share genetic risk factors and pathogenetic mechanisms with SLE. Individuals of Southern European or Asian ancestry have a greater likelihood of developing SLE,^1^ and those who do had increased drusen counts and larger drusen in this study. Pathogenetic mechanisms shared by both SLE and drusen are suggested by higher counts and larger drusen being associated with renal impairment and class IV lupus nephritis. Shared mechanisms are further suggested by the similar composition of drusen and immune deposits in GN, comprising complement, immunoglobulins, and extracellular matrix.^32^

Complement activation is important in both SLE and drusen development. The classical complement pathway predominates in SLE (*CFH, CFB, CFI, C3, C4, C2, C1q,r,s)*^41^ and the alternative pathway in drusen development.^24^ CFH is important in both: it is the major regulator of the alternative complement pathway but also inhibits C1q in the classical pathway. CFH clears cell debris^42^ but also protects against inappropriate complement activation in the extracellular matrix including on basement membranes in the glomerulus and retina.^43^ The major *CFH* drusen risk alleles for macular degeneration are Y402H^44^ and I62V^45^ in Europeans and Asians, respectively, and while they do not predispose to SLE, they may be associated with earlier onset lupus nephritis.^46^

Our observations suggest that abundant retinal drusen reflect the inflammatory burden within the glomerulus and correlate with renal impairment. A larger series may confirm the correlation with glomerular C3 deposits. In other forms of GN, renal damage is more severe with complement activation^47^ and is less severe in IgA and anti−glomerular basement membrane disease, where there are no glomerular complement deposits.^48^^,^^49^ Treatments targeting the mechanisms underlying drusen formation such as alternative complement pathway activation may also be effective in severe lupus nephritis. Already, severe proliferative lupus nephritis has been treated successfully with anti-C5 antibodies.^50^

The strengths of this study were its size and the rigor and reproducibility of the drusen assessments. Its major limitation was that retinal photographs were taken at different stages of disease.

Thus, drusen are common in SLE and may share pathogenetic mechanisms with SLE and lupus nephritis. Drusen may result from the retinal deposition of circulating immune complexes and local complement activation because they are also found in other forms of complement-mediated GN. Complement pathway genes have been implicated in drusen formation in macular degeneration and may also be involved in SLE-associated drusen.

## Disclosure

All the authors declared no competing interests.

## Supplementary Material

Supplementary File (PDF)

**STROB** **E Statement.**
