# Supplementary material for: Retinal Drusen Are More Common and Larger in Systemic Lupus Erythematosus With Renal Impairment
Source: Kidney Int Rep. 2022 Feb 2;7(4):848–56. doi: 10.1016/j.ekir.2022.01.1063 (PMC9039474; doi:10.1016/j.ekir.2022.01.1063)
Supplement: Supplementary File (PDF) [file mmc1.pdf]

Retinal drusen are more common and larger in SLE with renal impairment

STROBE Statement—checklist of items that should be included in reports of observational studies

	Item No.	Recommendation	
**Title and abstract**	1	(*a*) Indicate the study's design with a commonly used term in the title or the abstract	YES, p2.
		(*b*) Provide in the abstract an informative and balanced summary of what was done and what was found	YES, p2.
**Introduction**			
Background/rationale	2	Explain the scientific background and rationale for the investigation being reported	YES p3
Objectives	3	State specific objectives, including any prespecified hypotheses	YES p4
**Methods**			
Study design	4	Present key elements of study design early in the paper	YES, p5
Setting	5	Describe the setting, locations, and relevant dates, including periods of recruitment, exposure, follow-up, and data collection	YES, p5
Participants	6	(*a*) *Cohort study*—Give the eligibility criteria, and the sources and methods of selection of participants. Describe methods of follow-up<br>*Case-control study*—Give the eligibility criteria, and the sources and methods of case ascertainment and control selection. Give the rationale for the choice of cases and controls<br>*Cross-sectional study*—Give the eligibility criteria, and the sources and methods of selection of participants	YES, p5
		(*b*) *Cohort study*—For matched studies, give matching criteria and number of exposed and unexposed<br>*Case-control study*—For matched studies, give matching criteria and the number of controls per case	Case-control matched for age and gender
Variables	7	Clearly define all outcomes, exposures, predictors, potential confounders, and effect modifiers. Give diagnostic criteria, if applicable	YES, p5
Data sources/ measurement	8*	For each variable of interest, give sources of data and details of methods of assessment (measurement). Describe comparability of assessment methods if there is more than one group	YES, p5
Bias	9	Describe any efforts to address potential sources of bias	Pilot study

Study size	10	Explain how the study size was arrived at	Not applicable
Quantitative variables	11	Explain how quantitative variables were handled in the analyses. If applicable, describe which groupings were chosen and why	
Statistical methods	12	(*a*) Describe all statistical methods, including those used to control for confounding	YES, p6
		(*b*) Describe any methods used to examine subgroups and interactions	YES, p6
		(*c*) Explain how missing data were addressed	Numbers indicated
		(*d*) *Cohort study*—If applicable, explain how loss to follow-up was addressed<br>*Case-control study*—If applicable, explain how matching of cases and controls was addressed<br>*Cross-sectional study*—If applicable, describe analytical methods taking account of sampling strategy	Case – control – matched on basis of age and gender
		(*e*) Describe any sensitivity analyses	Not applicable
**Results**			
Participants	13*	(a) Report numbers of individuals at each stage of study—eg numbers potentially eligible, examined for eligibility, confirmed eligible, included in the study, completing follow-up, and analysed	Single point of recruitment
		(b) Give reasons for non-participation at each stage	Not relevant
		(c) Consider use of a flow diagram	Not relevant
Descriptive data	14*	(a) Give characteristics of study participants (eg demographic, clinical, social) and information on exposures and potential confounders	Table 1
		(b) Indicate number of participants with missing data for each variable of interest	Indicated in Tables
		(c) *Cohort study*—Summarise follow-up time (eg, average and total amount)	No follow up
Outcome data	15*	*Cohort study*—Report numbers of outcome events or summary measures over time	
		Case-control study—Report numbers in each exposure category, or summary measures of exposure	Not relevant
		Cross-sectional study—Report numbers of outcome events or summary measures	

Main results	16	(*a*) Give unadjusted estimates and, if applicable, confounder-adjusted estimates and their precision (eg, 95% confidence interval). Make clear which confounders were adjusted for and why they were included	P7-8, Table 1,2,3
		(*b*) Report category boundaries when continuous variables were categorized	
		(*c*) If relevant, consider translating estimates of relative risk into absolute risk for a meaningful time period	
Other analyses	17	Report other analyses done—eg analyses of subgroups and interactions, and sensitivity analyses	Table 2,3
**Discussion**			
Key results	18	Summarise key results with reference to study objectives	P9 – 10, Tables 1-3
Limitations	19	Discuss limitations of the study, taking into account sources of potential bias or imprecision. Discuss both direction and magnitude of any potential bias	P10
Interpretation	20	Give a cautious overall interpretation of results considering objectives, limitations, multiplicity of analyses, results from similar studies, and other relevant evidence	P9-10
Generalisability	21	Discuss the generalisability (external validity) of the study results	P10
**Other information**			
Funding	22	Give the source of funding and the role of the funders for the present study and, if applicable, for the original study on which the present article is based	No funding

*Give information separately for cases and controls in case-control studies and, if applicable, for exposed and unexposed groups in cohort and cross-sectional studies.

Note: An Explanation and Elaboration article discusses each checklist item and gives methodological background and published examples of transparent reporting. The STROBE checklist is best used in conjunction with this article (freely available on the Web sites of PLoS Medicine at http://www.plosmedicine.org/, Annals of Internal Medicine at http://www.annals.org/, and Epidemiology at http://www.epidem.com/). Information on the STROBE Initiative is available at www.strobe-statement.org.
